# Older Adults Interacting With Social Robots: Toward Greater Social Participation and Connection

**DOI:** 10.1093/geroni/igaf122.774

**Published:** 2025-12-31

**Authors:** Mélanie Levasseur, Mariam Fdil, Francois Michaud, Marika Lussier-Therrien, Dominic Létourneau

**Affiliations:** Université de Sherbrooke, Sherbrooke, Quebec, Canada; Université de Sherbrooke Faculty of Engineering and Interdisciplinary Institute for Technological Innovation, Sherbrooke, Quebec, Canada; Université de Sherbrooke, Sherbrooke, Quebec, Canada; Université de Sherbrooke Faculty of Medicine and Health Sciences, Sherbrooke, Quebec, Canada; Université de Sherbrooke Faculty of Engineering and Interdisciplinary Institute for Technological Innovation, Sherbrooke, Quebec, Canada

## Abstract

Some older adults require stimulation, learning, or assistance in their interactions, particularly when having disabilities. Social robots are promising to foster their participation and connection, and prevent situations of isolation. To our knowledge, Tabletop (T-Top) presents the most advanced communication and autonomous reasoning capabilities, but little is known about how older adults interact and perceived their experience with this robot. This study thus aimed to explore how older adults interact and perceive their experience with the social robot T-Top. An exploratory qualitative clinical research design was used with semi-directed interviews and observation of six older adults living in one senior residence. Older participants interacted with T-Top twice for about 30 minutes, an observation grid was used to identify their reactions and interactions, followed by interviews with a semi-structured guide to explore their experience and satisfaction. During the interaction sessions, all older adults expressed joy and surprise and maintained interested eye contact with the robot. The majority of older adults engaged in conversation with T-Top and actively participated. Although all participants appreciated the robot’s responsiveness and interactive behaviour, and, for most of them, its thinking and listening skills, some older adults were bothered by lack of perseverance and artificial appearance of T-Top. This study highlighted that social robots like T-Top should be personalized to older adults’ needs, preferences, and habits to facilitate interactions. More studies are needed to evaluate how such promising interventions can be used to foster older adults’ social participation and connection, and to prevent situations of isolation.

